# Diabetes prevalence by HbA1c and oral glucose tolerance test among HIV-infected and uninfected Tanzanian adults

**DOI:** 10.1371/journal.pone.0230723

**Published:** 2020-04-08

**Authors:** Kidola Jeremiah, Suzanne Filteau, Daniel Faurholt-Jepsen, Brenda Kitilya, Bazil B. Kavishe, Rikke Krogh-Madsen, Mette F. Olsen, John Changalucha, Andrea M. Rehman, Nyagosya Range, Jerome Kamwela, Kaushik Ramaiya, Aase B. Andersen, Henrik Friis, Douglas C. Heimburger, George PrayGod

**Affiliations:** 1 Mwanza Research Centre, National Institute for Medical Research, Mwanza, Tanzania; 2 Faculty of Epidemiology and Population Health, London School of Hygiene & Tropical Medicine, London, United Kingdom; 3 Department of Infectious Diseases, Rigshospitalet, Copenhagen, Denmark; 4 Centre of Inflammation and Metabolism and Centre for Physical Activity Research, Rigshospitalet, University of Copenhagen, Copenhagen, Denmark; 5 Department of Nutrition, Exercise and Sports, University of Copenhagen, Copenhagen, Denmark; 6 Muhimbili Medical Research Centre, National Institute for Medical Research, Dar es Salaam, Tanzania; 7 Tanzania Commission for AIDS, Dar es Salaam, Tanzania; 8 Hindu Mandal Hospital, Dar es Salaam, Tanzania; 9 Vanderbilt Institute for Global Health and Department of Medicine, Vanderbilt University Medical Center, Nashville, TN, United States of America; University of Pisa, ITALY

## Abstract

**Background:**

The burden of diabetes is increasing in sub-Saharan Africa, including among people living with HIV. We assessed the prevalence of diabetes and the roles of HIV, antiretroviral therapy (ART) and traditional risk factors among adults in Tanzania.

**Methods:**

We analysed diabetes-relevant baseline data from 1,947 adult participants in the CICADA study in Mwanza, Tanzania: 655 HIV-uninfected, 956 HIV-infected ART-naïve, and 336 HIV-infected persons on ART. WHO guidelines for haemoglobin A1c (HbA1c) and oral glucose tolerance test (OGTT) were used to define diabetes and prediabetes. Risk factors were evaluated using multinomial logistic regression analysis. Relative risk ratios (RRR) were generated comparing participants with diabetes and prediabetes against the reference of those with no diabetes.

**Results:**

Mean age was 41 (SD 12) years; 59% were women. The prevalence of diabetes was 13% by HbA1c and 6% by OGTT, with partial overlap among participants identified by the two tests. Relative to HIV-uninfected, HIV-infected ART-naïve persons had increased relative risks of diabetes (HbA1c: RRR = 1.95, 95% CI 1.25–3.03; OGTT: RRR = 1.90, 95% CI 0.96–3.73) and prediabetes (HbA1c: RRR = 2.89, 95% CI 1.93–4.34; OGTT: RRR = 1.61, 95% CI 1.22–2.13). HIV-infected participants on ART showed increased risk of prediabetes (RRR 1.80, 95% CI 1.09, 2.94) by HbA1c, but not diabetes. CD4 count < 200 cell/μL at recruitment increased risk and physical activity decreased risk of diabetes by both HbA1c and OGTT.

**Conclusions:**

The prevalence of diabetes was high, especially among HIV-infected ART-naïve adults. Being more physically active was associated with lower risk of diabetes. HbA1c and OGTT identified different participants as having diabetes or prediabetes. Overall, the finding of high burden of diabetes among HIV-infected persons suggests that health systems should consider integrating diabetes screening and treatment in HIV clinics to optimize the care of HIV patients and improve their health outcomes.

## Introduction

Diabetes mellitus is an emerging public health problem in sub-Saharan Africa (SSA) [1, 2], due to the nutrition transition and globalization [1, 3]. Increasing overall life expectancy in SSA further increases the risk of diabetes [4]. Compared to people from high-income countries, inhabitants of SSA seem to be at risk of diabetes at younger age and may have different risk factors including HIV infection [3, 5, 6]. Scarce data from SSA [7] limits health system responses to non-communicable diseases (NCDs) including diabetes [2].

Most research on diabetes in HIV-infected individuals has been done in high-income countries where data suggest that, although antiretroviral therapy (ART) can suppress viral load, the health of HIV-infected people is not completely restored, and people living with HIV have higher risk of diabetes than HIV-uninfected [8]. Studies investigating the mechanisms of glucose dysregulation among HIV-infected persons have been conflicting, but they mainly suggest that higher risk is related to HIV infection, co-morbidities, micronutrient deficiencies, specific antiretroviral drugs, individual genetic susceptibility or combinations of these factors [9, 10]. HIV infection triggers immune activation and chronic inflammation, which persist even after ART initiation, and this may lead to diabetes [11, 12]. During early HIV recovery in malnourished patients, rapid regain of fat rather than lean mass may occur [13], and could potentially increase the risk of diabetes. Although HIV and ART may have similar effects on diabetes in SSA as in high-income countries, such data cannot be directly extrapolated to SSA because of differing risk factors such as a lower prevalence of obesity, history of nutritional deficiencies, higher exposure to infections, and use of older antiretroviral drugs which have been associated with excess diabetes risk [14–17]. We therefore conducted a study to investigate the prevalence of diabetes and to assess the roles of HIV infection, ART and traditional risk factors.

## Methods

### Study design and setting

This was a cross-sectional study using baseline data (October 2016 to November 2017) from adults (age ≥18 years) enrolled in the Diabetes and Associated Complications in HIV Patients study which is locally called Chronic Infections, Co-morbidities and Diabetes in Africa (CICADA), a cohort study investigating the burden of and risk factors for diabetes in Mwanza, Tanzania, registered at https://clinicaltrials.gov as NCT03106480.

### Participant eligibility

Three cohorts were invited to join CICADA: (1) participants in Nutrition, Diabetes and Pulmonary Tuberculosis (TB-NUT), registered at https://clinicaltrials.gov/ as NCT00311298, conducted from 2006 to 2009. [6, 18–20]; (2) participants in Nutritional Support for African Adults Starting Antiretroviral Therapy (NUSTART), conducted from August 2011 to December 2013 [21], registered at the Pan African Clinical Trial registry as PACTR201106000300631; and (3) a new cohort comprising newly diagnosed HIV-infected, ART-naïve participants from ART clinics in Mwanza and HIV-uninfected controls from the neighbourhoods of new HIV cohort participants. The overall CICADA cohort thus comprised HIV-infected individuals on ART as well as HIV-infected ART-naïve individuals and HIV-uninfected controls.

### Recruitment of participants from previous cohorts

All participants who were known to be alive at the end of the follow-up in the previous cohorts (TB-NUT and NUSTART) were telephoned and introduced to the CICADA study. If participants were interested an appointment was scheduled. Participants who were not reachable by phone were visited at home and invited to join the study. If they were still not reachable, people responding to the phone call or those found in participants’ homes were requested to provide information on participants’ vital status and if they had travelled temporarily or relocated permanently from Mwanza. Eligible persons were requested to come to the research clinic in Mwanza at 8:00 am after an overnight fast of at least 8 hours to receive further study information, for consenting and for study procedures.

### Recruitment of new HIV-infected participants and HIV-negative controls

HIV-infected people who visited ART clinics in Mwanza City and were preparing to start ART were provided information about the study and asked to come to the study clinic for enrolment if they were aged 18 years and above, residents of Mwanza City, willing to consent and intending to remain in the area for the next three years. Using a computer-generated randomization list, we randomly took half of the new HIV-infected participants and selected HIV-uninfected participants for frequency matching. Criteria for HIV-negative participants were: lived within the same neighbourhood as the HIV index participant (defined as living in the same street or sub-village), HIV-negative based on HIV rapid tests, had lived in Mwanza City for at least 3 months with no plans for relocating within the next 3 years, aged 18 years or above and age difference with HIV-infected index participant not more than 5 years, same sex as the HIV-infected index participant, and willing to consent. Local street leaders of selected index case were requested to provide a list of households in their street, then 3 households were randomly chosen for eligibility criteria. If no one in these households met the criteria the process was repeated until a suitable household was found.

### Questionnaire data

Data were collected using structured interviews. Information was gathered on demography, education, occupation, religion, marital status and, using WHO STEPS questionnaire and show cards [22], on level of physical activity and alcohol use. For alcohol intake, we also used previously developed coloured show cards of commonly available alcoholic drinks in Mwanza [23], with their equivalent standard drink conversions. Information on history of ART use, adherence, smoking, TB treatment, and symptoms of diabetes were also collected. ART history was verified through participants’ ART cards or clinic records and grouped based on regimens containing zidovudine (AZT), abacavir (ABC), or tenofovir (TDF) because of indication of these drugs to have effect on glucose tolerance [24]. Total physical activity in metabolic equivalents in minutes per week (MET- minutes) was computed from the STEPS questionnaire and categorized as <600 or ≥ 600 MET-minutes based on the WHO recommendation [25].

### Diabetes measures

Two indicators of diabetes were used: glycated haemoglobin A1c (HbA1c), and 2-hour oral glucose tolerance test (OGTT). Participants were contacted one day prior to the clinic visit and instructed to come fasting. Upon arrival and before glucose testing, participants were asked if they had taken any food except water (i.e. fasted) for at least 8 hours before visiting the clinic. Those fasting were requested to provide venous blood for HbA1c and glucose measurement (Hemocue 201 RT, and Hemocue HbA1c 501 respectively; Hemocue AB, Angelholm, Sweden). They were then given 82.5g of dextrose monohydrate (equivalent to 75g of anhydrous glucose) diluted in 250 ml of drinking water to drink within 5 minutes for OGTT, and blood specimens were collected after 30 minutes and 2 hours. For this analysis we used OGTT data at 2 hours only. Those who reported not fasting were instructed to come fasting the next day for testing.

We used WHO guidelines [26] to categorize test results in three levels: normal, prediabetes, and diabetes mellitus: For OGTT, a 2-hour glucose level 7.8–11.0 mmol/L indicated prediabetes [27], and ≥11.1 mmol/L indicated diabetes; for HbA1c, prediabetes was 5.7–6.4% and diabetes was ≥6.5%. In addition, we looked at fasting plasma glucose (FPG) as a supplementary diabetic test for comparison purpose since FPG is widely used in clinical practice. (S1 Table).

### HIV and CD4 testing

Venous blood was collected for other tests including HIV status (for participants with unknown HIV status). HIV testing was done using two rapid antibody tests (SD HIV- 1/2 3.0 SD standard diagnostics Inc, and The Uni-Gold, Trinity Biotech, IDA Business Park, Bray, Co. Wicklow, Ireland). Discordant samples were tested using Uniform II vironostika-HIV Ag/Ab Micro-Elisa system (Biomerieuxbv, The Netherlands), and CD4 counts (cells/μL) using CyflowPartec machine (*Partec* GmbH, Munster, Germany).

### Anthropometry and body composition

Anthropometric measurements were assessed using standardized methods [28]. With participants barefoot and wearing minimal clothing, body weights were determined to the nearest 0.1 kg using a digital scale (Seca, Germany), and height was measured to the nearest 0.1 cm using a stadiometer fixed to the clinic wall (Seca, Germany). Mid-upper arm, waist, and hip circumferences were measured using non-stretchable tape (to 1 mm). Anthropometric measurements were taken in triplicate, and medians were used during analysis. Anthropometric data collectors were trained to acceptable proficiency before data collection commenced. Body mass index (BMI) was calculated as weight (kg)/height (m)^2^ and WHO cut-off values were used to classify participants as underweight (BMI <18.5kg/m^2^), normal weight (BMI 18.5–24.99 kg/m^2^), and overweight or obese (BMI ≥25 kg/m^2^). Waist circumferences ≥102 cm in males and ≥88 cm in females were considered elevated. Participants underwent bioelectrical impedance analysis (BIA) to estimate fat mass (kg) and fat-free mass (kg) using a body composition analyser (Tanita BC418, Tokyo, Japan). These body composition parameters were converted to fat mass index (FMI) and fat-free mass index (FFMI) by dividing their values by height (m)^2^. Handgrip strength was determined to the nearest 0.1 kg using a digital dynamometer (Takei Scientific Instruments, Japan). Four measurements were taken, with the mean of the two maximum measurements (one in each hand) reported.

### Ethics statement

Ethical permission to conduct the study was granted by the Medical Research Coordinating Committee (MRCC) of the National Institute for Medical Research (NIMR) in Tanzania, the London School of Hygiene and Tropical Medicine and consultative approval from the National Committee on Health Research Ethics in Denmark. Only patients giving informed consent were included. Oral and written information in Swahili were provided to all participants prior to obtaining informed oral and written consent.

### Data management and statistics

Data were entered into CSPro and analysed in STATA version 13. Descriptive analysis was done using histograms for shape distributions and the Shapiro Wilk test for normality for all continuous variables. Cohort characteristics are presented as medians and interquartile ranges, means and SDs, or percentages as appropriate. Chi-square tests were used to compare diabetes and prediabetes between HIV status groups. In addition to HIV status group, other conventional and novel risk factors for diabetes and prediabetes included in analyses were age, sex, smoking history, alcohol use, physical activity in metabolic equivalents (a computation which included combination of total time spent in moderate- and vigorous-intensity activity per week calculated based on WHO recommendation and categorized as <600 or ≥600 MET-minutes) [25], grip strength, fruit and vegetable intake, socioeconomic status (SES, calculated using principal component analysis of variables), BMI, FMI, FFMI, and TB history. SES, grip strength, FMI and FFMI were grouped in categorical tertiles, age into decades, and CD4 counts were divided into groups of <200, 200–500, and >500 cells/μL.

Univariate multinomial logistic regression models were done for all predictor variables with the outcomes, prediabetes and diabetes. The associations were presented as relative risk ratios (RRR) with 95% confidence intervals. Next, we conducted multivariable multinomial logistic regressions including as independent variables HIV status group, age, sex, FMI and FFMI plus any variables with an overall effect size of *p*<0·20 in the univariable analyses for that diabetes test. To avoid effects of multicollinearity, all predictor variables which qualified for inclusion in multivariable multinomial regression analysis were assessed for collinearity measured as Variance Inflation Factor (VIF). Those with VIF <5 were categorized as having no or moderate collinearity which did not warrant corrective measure and were included in the final models. BMI, FMI, and FFMI showed a degree of collinearity with VIF >5, so final models omitted BMI and included only its components, FMI and FFMI. Similarly, the original study from which participants came was omitted as a variable because of collinearity with TB history and ART.

## Results

**Fig 1:**
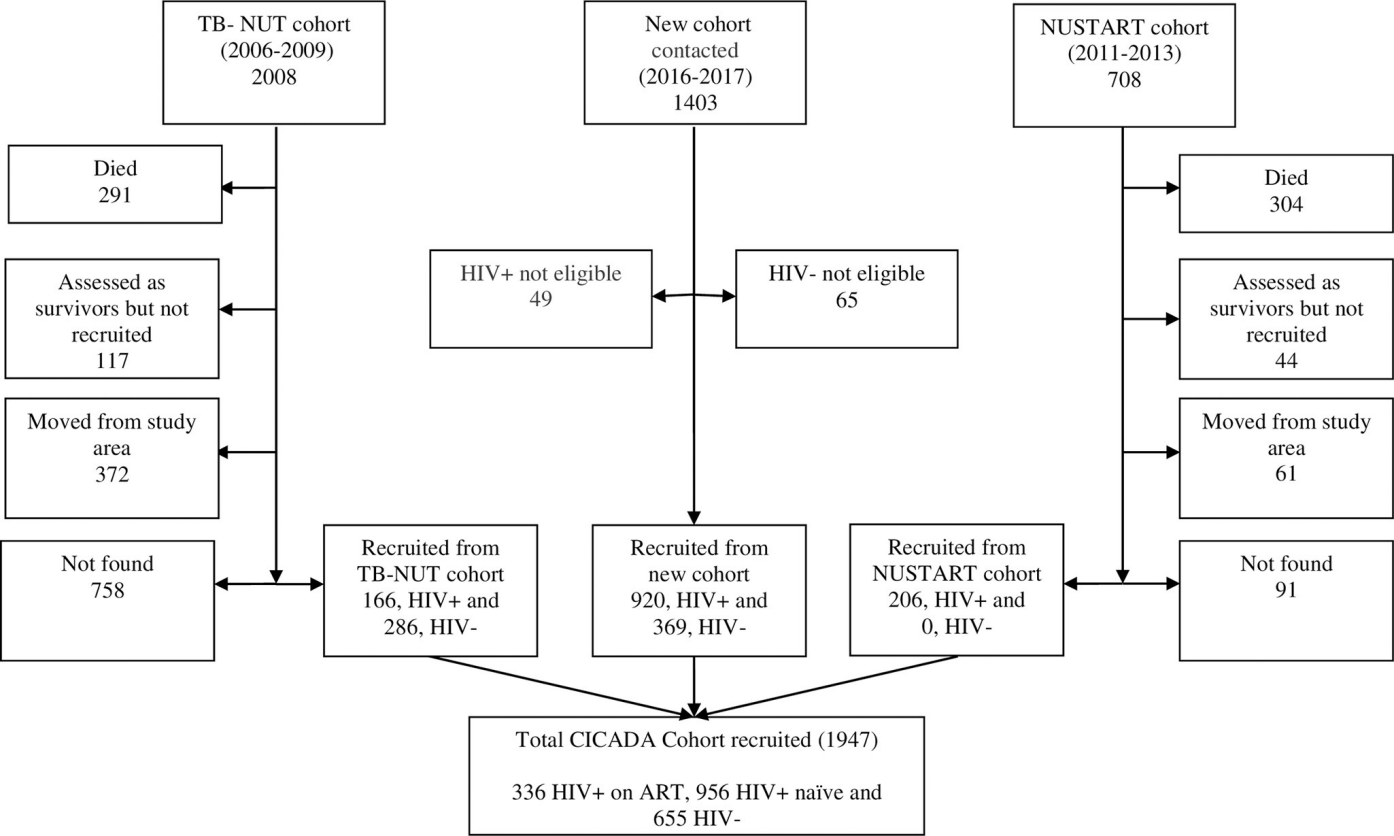
Flow chart showing source and composition of CICADA cohort. Acronyms: HIV+ = HIV-infected, HIV- = HIV-uninfected, HIV+ on ART = HIV infected on antiretroviral therapy, and HIV+ ART-naïve = HIV not on antiretroviral therapy.

Of 1,947 study participants, 1,157 (59%) were female. The mean age was 41 years (SD 12), 655 (34%) were HIV-uninfected, 956 (49%) were HIV-infected and ART-naïve, and 336 (17%) were HIV-infected on ART (Fig 1). There were 452 (22%) and 206 (29%) participants from the original TB-NUT and NUSTART cohorts respectively; loss of those originally enrolled in these previous trials was due to high mortality and loss to follow-up.

The median ART duration for those on ART was 53 months (IQR: 46; 102). Most of the HIV-uninfected participants were married or cohabiting but about half of the HIV-infected people were divorced/separated (Table 1). HIV-uninfected participants tended to be more educated and of higher SES and BMI than HIV-infected participants.

**Table 1:** Demographic and background characteristics of participants^1, 2, 3^.

Characteristics	N	HIV STATUS			P^4^
		HIV- n = 655	HIV+ ART- n = 956	HIV+ ART+ n = 336	
**Age**, years, mean (SD)	**1947**	42.2 (13.2)	38.2 (10.9)	44.9 (10.1)	<0.001
18–30		139 (21.2)	256 (26.7)	25 (7.4)	<0.001
31–40		183 (27.9)	342 (35.8)	98 (29.2)	
41–50		171 (26.1)	223 (23.3)	127 (37.8)	
>50		162 (24.7)	135 (14.1)	86 (25.6)	
**Sex**, female	**1947**	371 (56.6)	577 (60.4)	209 (62.2)	0.17
**Marital status**	**1942**				
Married/cohabiting		454 (69.3)	434 (45.6)	152 (45.4)	<0.001
Widowed/separated /divorced		140 (21.4)	448 (47.1)	172 (51.3)	
Single		61 (9.3)	70 (7.3)	11 (3.3)	
**Education level**	**1942**				
No formal education		80 (12.2)	158 (16.6)	81 (24.2)	<0.001
Primary education		426 (65.0)	676 (71.0)	224 (66.8)	
Secondary/tertiary college		149 (22.8)	118 (12.4)	30 (9.0)	
**Employment**	**1942**				
Salaried		62 (9.5)	165 (17.3)	41 (12.3)	<0.001
Self-employed		483 (73.7)	654 (68.7)	256 (76.4)	
Unemployed/housewife		110 (16.8)	133 (14.0)	38 (11.3)	
**Ever treated for TB**	**1945**	526 (80.3)	897 (94.0)	147 (43.7)	<0.001
**Socioeconomic status**	**1942**				
Lower		167 (25.5)	316 (33.2)	165 (49.3)	<0.001
Middle		220 (33.6)	328 (34.5)	99 (29.5)	
Upper		268 (40.9)	308 (32.3)	71 (21.2)	
**Fruit and vegetable consumption (servings/day)**	**1929**				<0.001
0		74 (11.4)	148 (15.7)	17 (5.1)	
1–2		368 (56.4)	494 (52.4)	152 (45.4)	
3–4		135 (20.7)	196 (20.8)	89 (26.6)	
≥ 5		75 (11.5)	104 (11.0)	77 (22.9)	
**Physical activity (MET-minutes per week)**^5^	**1941**				
< 600		82 (12.6)	137 (14.4)	51 (15.2)	0.44
≥600		571 (87.4)	815 (85.6)	285 (84.8)	
**Smoking**					
Never		507 (77.5)	727 (76.4)	240 (71.4)	<0.001
Ex-smoker		89 (13.6)	116 (12.2)	81 (24.1)	
Current smoker		58 (8.9)	109 (11.4)	15 (4.5)	
**Never taken alcohol**	**1942**	212 (32.4)	248 (26.1)	93 (16.8)	0.02
**Grip strength (kg)**, mean (SD)^6^	**1945**	30.3 (8.7)	26.5 (8.4)	28.3 (8.4)	0.54
**Body mass index (kg/m**^**2**^**)**, mean (SD)	**1946**	23.6 (4.9)	21.1 (4.1)	20.7 (3.7)	<0.001
<18.5 kg/m^2^		86 (13.1)	254 (26.6)	86 (25.6)	<0.001
18.5–25.0 kg/m^2^		351 (53.6)	565 (59.2)	214 (63.7)	
> 25.0 kg/m^2^		218 (33.3)	136 (14.2)	36 (10.7)	
**Waist circumference** (cm), mean (SD)^7^	**1946**	83.5 (12.6)	77.0 (9.5)	78.6 (9.9)	<0.001
Normal		500 (76.3)	863 (90.4)	290 (86.3)	<0.001
Increased		155 (23.7)	92 (9.6)	46 (13.7)	
**Fat mass index (kg/m**^**2**^**)**, mean (SD)^8^	**1900**	6.3 (3.9)	4.6 (3.4)	4.5 (3.1)	<0.001
Lower tertile		153 (23.6)	360 (39.1)	121 (36.7)	<0.001
Middle tertile		202 (31.1)	299 (32.5)	132 (40.0)	
Upper tertile		294 (45.3)	262 (28.5)	77 (23.3)	
**Fat-free mass index**, (kg/m^2^) mean (SD)^9^	**1900**	17.1 (1.9)	16.3 (1.8)	16.0 (1.6)	0.002
Lower tertile		143 (22.0)	345 (37.5)	146 (44.2)	<0.001
Middle tertile		216 (33.3)	301 (32.7)	116 (35.2)	
Upper tertile		290 (44.7)	275 (29.8)	68 (20.6)	
**CD4 count** (cell/μL), median (IQR)	**1944**	678 (542; 831)	224 (93; 401)	366 (242; 488)	0.01
More than 500		534 (81.8)	152 (15.9)	79 (23.5)	<0.001
200–500		113 (17.3)	366 (38.3)	204 (60.7)	
Less than 200		6 (0.9)	437 (45.7)	53 (15.8)	
**Duration on ART** (Months), median (IQR)	**333**	0	0	53 (47; 102)	
**Cohort recruited**	**1947**				
TB-NUT cohort		286 (43.7)	37 (3.9)	129 (38.4)	<0.001
NUSTART cohort		0 (0.0)	5 (0.5)	201 (59.8)	
New cohort		369 (56.3)	914 (95.6)	6 (1.8)	

^1^Data are number (%) unless specifically indicated as mean (SD) or median (IQR);

^2^ Data do not sum to 1947 due to missing values;

^3^ HIV- = HIV uninfected, HIV+ ART- = HIV infected not on ART, HIV+ART+ = HIV on ART

^4^X^2^– test used for categorical variables, A one-way analysis of variance for parametric variables and Kruskal Wallis for non-parametric variables

^5^Computation included combination of total time spent in moderate and in vigorous physical activity per week

^6^Grip strengths tertile cut offs defined as; 0.0–23.6 for lower; 23.7–30.6 for middle and 30.7–59.6 for upper

^7^Waist circumference increase is defined as ≥88 cm for women and ≥ 102 cm for men

^8^Fat mass index tertile cut offs defined as; 0.19–3.03 for lower; 3.04–5.76 for middle and 5.77–22.00 for upper

^9^Fat-free mass index tertile cut offs defined as; 11.12–15.61 for lower; 15.62–17.20 for middle and 17.21–24.24 for upper

The prevalence of diabetes and prediabetes differed by the two tests and across HIV status (Table 2). The overall prevalence of diabetes was 13% by HbA1c, and 6% by OGTT. The tests identified overlapping but not identical groups of participants (Table 2); in all groups, diabetes prevalence was higher by HbA1c than by OGTT, but prediabetes was higher by OGTT.

**Table 2:** Prevalence of prediabetes and diabetes as defined by oral glucose tolerance test (OGTT) and glycated haemoglobin A1c (HbA1c)^1, 2, 3^.

Characteristics	N	HIV-	HIV+, ART-	HIV+, ART+	P
**HbA1c (%)**	**1944**				
Normal (≤6)		536 (81.8)	567 (59.4)	259 (77.3)	<0.001
Prediabetes (6.0–6.4)		58 (8.9)	218 (22.9)	50 (14.9)	
Diabetes (≥6.5)		61 (9.3)	169 (17.7)	26 (7.8)	
**OGTT (mmol/L)**	**1941**				
Normal (≤7.7)		378 (58.1)	418 (43.8)	173 (51.6)	<0.001
Prediabetes (7.8–11.0)		247 (37.9)	450 (47.1)	151 (45.1)	
Diabetes (≥11.1)		26 (4.0)	87 (9.1)	11 (3.3)	
**HbA1c (%) and OGTT (mmol/L) overlap**	**1940**				
No diabetes or prediabetes by any measure		581 (89.3)	731 (76.6)	301 (89.8)	<0.001
Diabetes by HbA1c only		44 (6.7)	136 (14.3)	23 (6.9)	
Diabetes by OGTT only		11 (1.7)	54 (5.6)	8 (2.4)	
Diabetes by both measures		15 (2.3)	33 (3.5)	3 (0.9)	

^1^Data are number (%)

^2^For each test, data do not sum to 1947 due to missing values

^3^HIV- = HIV uninfected, HIV+ ART- = HIV infected not on ART, HIV+ART+ = HIV infected on ART, X^2^– test used to test relationships between categorical variables

Table 3 shows risk factors for prediabetes and diabetes as assessed by HbA1c. Based on final multinomial multivariate models as described in the methods, compared with HIV-uninfected participants, being HIV-infected but not on ART was associated with increased risk of having diabetes (RRR = 1·95 [95%CI, 1·25–3.03]) and prediabetes (RRR = 2·89 [95%CI, 1·93–4.34]); being HIV-infected and on ART was associated with increased risk of prediabetes (RRR = 1.80 [95%CI, 1·09–2.94]) but not diabetes. Having CD4 count less than 200 cells/μl at recruitment was associated with increased risk of diabetes (RRR = 1.79 [95%CI, 1·13–2.83]) but not prediabetes. Having upper tertile of FFMI was associated with increased risk of diabetes (RRR = 1·66 [95%CI, 1·09–2.52]). FMI was not associated with diabetes or prediabetes. Factors associated with decreased risk of diabetes were greater than 600 metabolic equivalent-minutes of physical activity (RRR = 0·57 [95%CI, 0·0.39–0·83]) and upper tertile of grip strength. History of TB treatment was associated with reduced risk of diabetes and prediabetes in univariate analysis, but this did not hold up in multivariate analysis.

**Table 3:** Multinomial analysis of factors associated with prediabetes and diabetes assessed by glycated haemoglobin A1c (HbA1c), taking "no diabetes" as the reference category.

	Univariate^1^				Multivariate^1^			
	Prediabetes^2^ (N = 326)	P	Diabetes^2^ (N = 256)	P	Prediabetes^2^ (N = 326)	P	Diabetes^2^ (N = 256)	P^3^
	RRR, (95% CI)		RRR, (95% CI)		RRR, (95% CI)		RRR (95% CI)	
**Age (years)**								
18–30	1	0.01	1	0.89	1	0.50	1	0.36
31–40	1.05 (0.76; 1.44)		0.96 (0.65; 1.40)		1.05 (0.75; 1.47)		0.97 (0.64; 1.45)	
41–50	0.79 (0.55; 1.10)		1.04 (0.71; 1.54)		0.89 (0.61; 1.29)		1.19 (0.78; 1.81)	
>50	0.60 (0.40; 0.89)		1.11 (0.74; 1.67)		0.77 (0.49; 1.21)		1.37 (0.87; 2.18)	
**Sex**								
Female	1	<0.001	1	0.37	1	0.007	1	0.07
Male	**0.50 (0.38; 0.65)**		0.88 (0.67; 1.16)		**0.51 (0.31; 0.83)**		0.62 (0.37; 1.03)	
**HIV status**								
Negative	1	<0.001	1	<0.001	1	<0.001	1	0.002
Positive not on ART	**3.55 (2.59; 4.86)**		**2.61 (1.91; 3.59)**		**2.89 (1.93; 4.34)**		**1.95 (1.25; 3.03)**	
Positive on ART	**1.78 (1.18; 2.67)**		0.88 (0.54; 1.43)		**1.80 (1.09; 2.94)**		0.99 (0.55; 1.76)	
**TB treatment history**								
Never treated for TB	1	0.004		<0.001	1	0.86	1	0.67
Treated for TB	**0.61 (0.44; 0.86)**		**0.42 (0.27; 0.64)**		0.96 (0.65; 1.43)		0.64 (0.39; 1.03)	
**Fruit and vegetable consumption (servings/day)**								
0	1	0.56	1	0.69	-		-	
1–2	0.81 (0.56; 1.18)		0.95 (0.63; 1.44)		-		-	
3–4	0.94 (0.62; 1.43)		0.82 (0.51; 1.34)		-		-	
≥ 5	0.98 (0.62; 1.57)		1.09 (0.65; 1.83)		-		-	
**CD4 count (cell/μL)**								
More than 500	1	<0.001	1	<0.001	1	0.007	1	0.001
200–500	1.29 (0.96; 1.75)		1.02 (0.72; 1.43)		0.84 (0.58; 1.20)		0.80 (0.53; 1.04)	
Less than 200	**2.84 (2.09; 3.84)**		**2.99 (2.17; 4.14)**		1.42 (0.95; 2.13)		**1.79 (1.13; 2.83)**	
**BMI (kg/m**^**2**^**)**								
18.5–25	1	<0.035	1	0.005	-		-	
<18.5	**1.38 (1.03; 1.86)**		**1.66 (1.20; 2.28)**		-		-	
> 25.0	**1.37 (1.01; 1.87)**		1.39 (0.98; 1.95)		-		-	
**Grip strength**^4^								
Lower tertile	1	<0.001	1	0.003	1	0.66	1	0.08
Middle tertile	**0.71 (0.53; 0.94)**		**0.63 (0.46; 0.87)**		0.89 (0.65; 1.21)		0.73 (0.51; 1.04)	
Upper tertile	**0.42 (0.31; 0.58)**		**0.52 (0.38; 0.73)**		0.83 (0.54; 1.28)		**0.61 (0.38; 0.97)**	
**Waist circumference (cm)**^5^								
Normal	1	0.21	1	0.21	-	-	-	
Increased	1.23 (0.89; 1.71)		1.26 (0.88; 1.79)		-	-	-	
**Fat mass index (kg/m**^**2**^**)**^6^								
Lower tertile	1	0.62	1	0.25	1	0.53	1	0.17
Middle tertile	1.07 (0.78; 1.45)		0.76 (0.54; 1.06)		0.82 (0.57; 1.69)		0.69 (0.47; 1.03)	
Upper tertile	1.16 (0.86; 1.57)		0.83 (0.60; 1.16)		0.90 (0.60; 1.36)		0.72 (0.46; 1.14)	
**Fat free mass index (kg/m**^**2**^**)**^7^								
Lower tertile	1	0.004	1	0.93	1	0.96	1	0.06
Middle tertile	0.79 (0.59; 1.06)		0.96 (0.68; 1.34)		1.02 (0.74; 1.41)		1.33 (0.92; 1.92)	
Upper tertile	0.59 (0.43; 0.80)		1.01 (0.73; 1.41)		1.06 (0.72; 1.55)		**1.66 (1.09; 2.52)**	
**Physical activity (MET-min)**^8^								
< 600	1	0.89	1	0.005	1	0.22	1	0.004
≥600	0.97 (0.68; 1.39)		**0.61 (0.43; 0.86)**		0.78 (0.52; 1.16)		**0.57 (0.39; 0.83)**	
**History of alcohol drinking**								
No	1	0.87	1	0.21	-		-	
Yes	0.98 (0.75; 1.28)		1.03 (0.76; 1.39)		-		-	
**Smoking history**								
Never	1	<0.004	1	0.91	1	0.34	1	0.39
Ex-smoker	**0.58 (0.39; 0.85)**		1.05 (0.72; 1.51)		0.84 (0.52; 1.35)		1.31 (0.84; 2.04)	
Current smoker	**0.58 (0.36; 0.94)**		1.09 (0.70; 1.69)		0.65 (0.36; 1.17)		0.92 (0.53; 1.61)	
**ART regimen**^9^								
AZT backbone	1	0.16	1	0.56	-		-	-
ABC and other backbone	1.05 (0.28; 3.89)		1.26 (0.26; 6.18)		-		-	-
TDF backbone	1.83 (0.96; 3.47)		1.59 (0.68; 3.72)		-		-	**-**
**Cohort recruited**								
TB -NUT cohort	1	<0.001	1	<0.001	-		-	-
NUSTART cohort	**1.75 (1.03; 2.98)**		1.14 (0.62; 2.09)		-		-	-
New cohort	**3.37 (2.33; 4.88)**		**2.69 (1.84; 3.93)**		-		-	-

^1^ Multivariate analysis included age, sex, fat mass index and fat free mass index; and all variables with overall P value <0.2 in the in univariate analyses; body mass index and cohort recruited were not included in multivariable analysis because of collinearity with other included variables.

^2^Cutoff point for defining diabetes and prediabetes based on WHO criteria; RRR = Relative risk ratio;

^3^ Bolded estimates within variables categories has P value <0.05

^4^Grip strengths tertile cut offs defined as; 0.0–23.6 for lower; 23.7–30.6 for middle and 30.7–59.6 for upper

^5^Waist circumference increase is defined as ≥88 cm for women and ≥ 102 cm for men

^6^Fat mass index tertile cut offs defined as; 0.19–3.03 for lower; 3.04–5.76 for middle and 5.77–22.00 for upper

^7^Fat-free mass index tertile cut offs defined as; 11.12–15.61 for lower; 15.62–17.20 for middle and 17.21–24.24 for upper

^8^Computation included combination of total time spent in moderate and in vigorous physical activity per week

^9^AZT- azidothymidine, ABC-Abacavir and TDF-Tenofovir containing regimen

When measured by OGTT (Table 4), compared to HIV-uninfected participants, being HIV-infected but not on ART was associated with increased risk of prediabetes (RRR = 1.61 [95%CI, 1·22–2.13]) and of diabetes (RRR = 1.90 [95%CI, 0.96–3.73,]). People who were HIV-infected and on ART had no differences in multivariable analyses in risk of diabetes or prediabetes compared to uninfected controls. CD4 count <200 cells/μl was associated with increased risk of diabetes (RRR = 2.71 [95%CI, 1.36–5.38] but not prediabetes. Adequate physical activity, upper tertile of grip strength and being a current smoker were associated with decreased risk of diabetes or prediabetes, while FMI and FFMI were not associated with diabetes or prediabetes. Older age and being male increased risk of diabetes.

**Table 4:** Multinomial analysis of factors associated with prediabetes and diabetes assessed by oral glucose tolerance test (OGTT), taking "no diabetes" as the reference category.

	Univariate^1^				Multivariate^1^			
	Prediabetes^2^ (N = 326)	P	Diabetes^2^ (N = 256)	P	Prediabetes^2^ (N = 326)	P	Diabetes^2^ (N = 256)	P^3^
	RRR, (95% CI)		RRR, (95% CI)		RRR, (95% CI)		RRR (95% CI)	
**Age (years)**								
18–30	1	0.03	1	<0.001	1	0.055	1	0.002
31–40	1.15 (0.89; 1.48)		1.51 (0.79; 2.85)		1.18 (0.90; 1.54)		1.44 (0.73; 2.85)	
41–50	1.28 (0.97; 1.66)		**2.24 (1.19; 4.21)**		**1.36 (1.02; 1.81)**		**2.36 (1.19; 4.64)**	
>50	**1.53 (1.14; 2.04)**		**4.08 (2.19;7.61)**		**1.52 (1.10; 1.11)**		**4.01 (1.99; 8.04)**	
**Sex**								
Female	1	0.005	1	0.001	1	0.62	1	0.03
Male	1.10 (0.91; 1.32)		**1.94 (1.33; 2.83**)		1.09 (0.79; 1.55)		**2.12 (1.07; 4.17)**	
**HIV status**								
Negative	1	<0.001	1	<0.001	1	0.002	1	0.004
Positive not on ART	**1.65 (1.34; 2.02)**		**3.03 (1.91; 4.79)**		**1.61 (1.22; 2.13)**		1.90 (0.96; 3.73)	
Positive on ART	**1.33 (1.02; 1.75)**		0.92 (0.44; 1.91)		1.19 (0.87; 1.65)		0.58 (0.24; 1.39)	
**TB treatment history**								
Never treated for TB	1	0.80	1	0.28	-		-	
Treated for TB	1.03 (0.82; 1.30)		0.75 (0.45; 1.26)		-		-	
**Fruit and vegetable consumption (servings/day)**								
0	1	0.07	1	0.61				
1–2	1.34 (0.99; 1.80)		1.21 (0.66; 2.22)		-		-	
3–4	1.40 (1.00; 1.95)		1.29 (0.65; 2.55)		-		-	
≥ 5	1.63 (1.13; 2.37)		1.62 (0.78; 3.37)		-		-	
**CD4 count (cell/μL)**								
More than 500	1	<0.001	1	<0.001	1	0.33	1	0.004
200–500	**1.25 (1.01; 1.54)**		1.08 (0.63; 1.83)		1.04 (0.80; 1.35)		0.98 (0.51; 1.89)	
Less than 200	**1.69 (1.33; 2.14)**		**4.35 (2.75; 6.83)**		1.24 (0.90; 1.71)		**2.71 (1.36; 5.38)**	
**BMI (kg/m**^**2**^**)**								
18.5–25	1	0.13	1	0.004	-		-	
<18.5	1.24 (0.98; 1.57)		**2.39 (1.55;3.67)**		-		-	
> 25.0	1.17 (0.92; 1.48)		1.41 (0.86;2.32)		-		-	
**Grip strength**^4^								
Lower tertile	1	0.27	1	0.08	1	0.64	1	0.02
Middle tertile	0.83 (0.66; 1.04)		0.69 (0.44; 1.08)		0.89 (0.70; 1.14)		0.71 (0.42; 1.19)	
Upper tertile	0.88 (0.70; 1.10)		**0.61 (0.38; 0.97)**		0.88 (0.64; 1.21)		**0.39 (0.20; 0.76)**	
**Waist circumference (cm)**^5^								
Normal	1	0.68	1	0.34	-		-	
Increased	1.05 (0.82; 1.36)		0.85 (0.48; 1.47)		-		-	
**Fat mass index (kg/m**^**2**^**)**^6^								
Lower tertile	1	0.72	1	0.009	1	0.78	1	0.51
Middle tertile	0.92 (0.73; 1.16)		**0.59 (0.37; 0.92)**		0.93 (0.72; 1.21)		0.74 (0.43; 1.25)	
Upper tertile	0.92 (0.73; 1.16)		**0.51 (0.32; 0.82)**		1.01 (0.74; 1.37)		0.88 (0.46; 1.68)	
**Fat free mass index (kg/m**^**2**^**)**^7^								
Lower tertile	1	0.67	1	0.05	1	0.74	1	0.21
Middle tertile	0.91 (0.73; 1.15)		0.56 (0.34; 0.91)		1.02 (0.79; 1.30)		0.71 (0.41; 1.20)	
Upper tertile	1.03 (0.79; 1.26)		0.91 (0.59; 1.42)		1.11 (0.84; 1.45)		1.11 (0.63; 1.97)	
**Physical activity (MET-min)**^8^								
< 600	1	<0.001	1	<0.001	1	<0.001	1	0.001
≥600	0.49 (0.37; 0.66)		**0.26 (0.26; 0.58)**		**0.53 (0.38; 0.71)**		**0.43 (0.19; 0.95**)	
**History of alcohol drinking**								
No	1	0.97	1	0.26	-		-	
Yes	1.00 (0.82; 1.23)		0.79 (0.53; 1.18)		-		-	
**Smoking history**								
Never	1	0.19	1	0.045	1	0.06	1	0.08
Ex-smoker	1.14 (0.87; 1.49)		**1.79 (1.12; 2.87)**		0.95 (0.69; 1.29)		1.06 (0.59; 1.89)	
Current smoker	0.79 (0.58; 1.10)		0.94 (0.48; 1.82)		**0.63 (0.43; 0.93)**		**0.43 (0.19; 0.94)**	
**ART regimen**^9^								
AZT backbone	1	0.19	1	0.43	-		-	
ABC and other backbone	1.32 (0.55; 3.29)		2.93 (0.51; 16.83)		-		-	
TDF backbone	1.52 (0.96; 2.40)		0.96 (0.24; 3.72)		-		-	
**Cohort recruited**								
TB -NUT cohort	1	0.69	1	0.10	-		-	
NUSTART cohort	1.12 (0.80; 1.57)		0.52 (0.21; 1.29)		-		-	
New cohort	0.98 (0.78; 1.23)		1.24 (0.78; 1.97)		-		-	

^1^ Multivariate analysis included age, sex, fat mass index and fat free mass index; and all variables with overall P value <0.2 in the in univariate analyses; body mass index and cohort recruited were not included in multivariable analysis because of collinearity with other included variables. ^2^Cutoff point for defining diabetes and prediabetes based on WHO criteria; RRR = Relative risk ratio;

^3^ Bolded estimates within variables categories has P value <0.05

^4^Grip strengths tertile cut offs defined as; 0.0–23.6 for Lower; 23.7–30.6 for Middle and 30.7–59.6 for Upper

^5^Waist circumference increase is defined as ≥88 cm for women and ≥ 102 cm for men

^6^Fat mass index tertile cut offs defined as; 0.19–3.03 for Lower; 3.04–5.76 for Middle and 5.77–22.00 for Upper

^7^Fat-free mass index tertile cut offs defined as; 11.12–15.61 for Lower; 15.62–17.20 for Middle and 17.21–24.24 for Upper

^8^Computation included combination of total time spent in moderate and in vigorous physical activity per week

^9^AZT- azidothymidine, ABC-Abacavir and TDF-Tenofovir containing regimen

## Discussion

We report a high burden of diabetes among Tanzanian adults, with an increased risk among HIV-infected individuals. This contrasts with recent systematic reviews suggesting that in the African setting, HIV and ART are not associated with diabetes, although the reviews’ findings may be limited by heterogeneity and small numbers of studies [7, 29]. Although diabetes prevalence varied with the different diagnostic tests, HIV positivity, especially without ART, was generally associated with increased risk of diabetes or prediabetes. For both OGTT and HbA1c tests, similar other risk factors, e.g. low CD4 count and low physical activity, were associated with increased risk of diabetes. The high prevalence estimates for diabetes found in our study are similar to those reported previously among Tanzanians with HIV infection [30, 31]. The observed variations in diabetes prevalence measured by the two tests have been reported elsewhere, and are likely related to the different components of the glucose metabolic pathway measured by the tests [32, 33].

Diabetes, particularly Type 2, results from insulin resistance and failure of beta-cell function, with the former preceding the latter [34]. Patients with prediabetes (impaired fasting glucose (IFG) and impaired glucose tolerance (IGT)), a transitional state of dysglycaemia preceding diabetes diagnosis by OGTT, have different degrees of both defects (beta-cell function failure and insulin resistance) which may occur together [35]. However, patients with IGT diagnosed by OGTT are more likely to have a defect in skeletal muscle insulin resistance (characterized by inability of skeletal muscle to utilize glucose). Unlike OGTT, Hb1Ac measures average glucose levels in the past 12 weeks and therefore is likely to diagnose patients with both marked hepatic and skeletal insulin resistance [36, 37].

Our finding that HIV infection is a risk factor for diabetes diagnosed by HbA1c and OGTT in adults not on ART may indicate that before starting ART, HIV-infected patients have high levels of muscle insulin resistance, probably resulting from inflammation and immune activation [11]. Although diabetes or prediabetes risk persists during ART, our observation that it is only apparent by HbA1c suggests that during ART, hepatic insulin resistance may be a driver of hyperglycemia, possibly due to liver fat accumulation or other adverse effects of ART drugs [38, 39]. Future work should focus on understanding the relative contributions of beta-cell deficiency and insulin resistance in the evolution of diabetes in HIV patients and how this affects the diagnostic characteristics of HbA1c and OGTT.

In our analysis, ageing, being male, and CD4 counts <200 cells/μL were significantly associated with diabetes by one or both tests. Ageing is well documented as a risk factor for diabetes, and male sex has been reported as a risk factor for diabetes among HIV-infected patients [40], although there is no clear explanation for this. In contrast to other studies showing increased diabetes risk with increased fat, especially central fat, we found virtually no associations of BMI, FMI, FFMI or waist circumference with diabetes risk. This could be because in general the population had normal or low BMI, with only 120 (6%) of the participants having BMI >30; virtually all of these were women and most were HIV-negative, so in multivariable analyses including sex and HIV status, BMI would have made limited additional contribution. We suspect that the increased risk of diabetes by HbA1c in the highest FFMI tertile may be a spurious association, especially since there was no evidence of increased risk in the univariable analyses.

The association of CD4 count with diabetes suggests that immune impairment resulting from HIV infection may magnify the risk of glucose dysregulation. Low CD4 count has previously been associated with diabetes in HIV-infected individuals [41] and helps explain the links between infectious and non-communicable diseases. Physical activity greater than 600 metabolic equivalents per week and an upper tertile of handgrip strength in our study were associated with reduced risk of diabetes. Physical activity tends to reduce fat storage and systemic inflammation and modulates metabolic balance [42]. Grip strength is a measure of muscular strength and good health in general, which may help reduce NCDs including diabetes. Individuals who are more active are less likely to have high blood pressure, type 2 diabetes, and metabolic syndrome [43]. However, given the cross-sectional nature of our study, it is possible that higher activity level merely reflects healthier people with no diabetes and better HIV control, which enable them to participate in work or leisure activities. An intervention trial would be needed to determine whether encouraging HIV-infected African adults to increase physical activity will reduce chances of developing diabetes.

Our finding that self-reported current smoking was associated with reduced risk of diabetes as assessed by OGTT is inconsistent with other studies reported elsewhere, where smoking was associated with diabetes [44]. Smoking may be a SES factor associated with being better off, but our finding may be spurious and related to a short time exposure between smoking initiation and being enrolled in our study, or bias with self-reporting, or that the majority who responded as current smokers were healthier. However, in the general population, studies have shown no differences in smoking behavior among those with and without diabetes [45].

A strength of our study was that it had a relatively large sample size to examine associations between risk factors and diabetes. In addition, unlike previous reports which used one method of diabetes diagnosis, we used two methods. This enabled us to examine associations among patients who may have different diabetes profiles, thus increasing our understanding of the roles of hypothesized risk factors in diabetes.

A limitation of our study is that patients on ART came from our previous TB-NUT and NUSTART cohorts which had high mortality, and it is likely that the observed risk represents diabetes risk among survivors. Furthermore, NUSTART recruited patients starting ART when malnourished (BMI <18.5 kg/m^2^), and our previous follow-up of the NUSTART participants [31] as well as other studies from Africa [46, 47] have suggested that malnutrition could lead to diabetes after nutritional recovery. Therefore, in our study it is hard to disentangle potential effects of ART and prior malnutrition on diabetes risk. Further longitudinal follow-up of HIV-infected persons newly enrolled in CICADA will be informative, as they will likely have lower HIV-related mortality risk because they started ART when healthier in comparison to those from our previous cohorts who were recruited when only those with low CD4 counts were offered ART. Higher survival rates in current cohorts treated for HIV when healthier, *i*.*e*., before becoming severely immunocompromised and developing AIDS, may result in different diabetes-related outcomes. Finally, this was a cross-sectional study, and we cannot conclude whether HIV and/or ART leads to diabetes.

## Conclusion

We found the prevalence of prediabetes or diabetes among HIV-infected persons to be higher than in HIV-uninfected persons, particularly for those not on ART. The relative merits of the different measures of prediabetes and diabetes among HIV-infected persons may require monitoring of end-organ impacts of diabetes over time. High-level physical activity was strongly associated with reduced risk of diabetes; if a causal association is confirmed by other studies, promoting physical activity among HIV-infected Africans may be important in reducing diabetes risk. Health systems should consider integrating diabetes screening and treatment in HIV care systems to optimize the care of HIV patients and improve their health outcomes.

## Supporting information

S1 Table Prevalence of prediabetes and diabetes as defined by fasting plasma glucose. (DOC)
