# Supplementary material for: Diabetes prevalence by HbA1c and oral glucose tolerance test among HIV-infected and uninfected Tanzanian adults
Source: PLoS One. 2020 Apr 8;15(4):e0230723. doi: 10.1371/journal.pone.0230723 (PMC7141607; doi:10.1371/journal.pone.0230723)
Supplement: S1 Table — (DOC) [file pone.0230723.s001.doc]

| **S1 Tables. Prevalence of prediabetes and diabetes as defined by fasting plasma glucose.**^1^**,** ^2^, ^3^ | | | | | |
| --- | --- | --- | --- | --- | --- |
| **Characteristics** |  |  | | | **P** |
|  | **N** | **HIV-** | **HIV+, ART-** | **HIV+, ART+** |  |
| **Fasting plasma glucose (mmol/L)** | **1945** |  |  |  |  |
| Normal (≤6.0) |  | 231 (35.3) | 290 (30.4) | 67 (20.0) | <0.001 |
| Prediabetes (6.1-6.9) |  | 247 (37.7) | 452 (47.3) | 159 (47.5) |  |
| Diabetes (≥7) |  | 177 (27.0) | 213 (22.3) | 109 (33.5) |  |
| ^1^Data are number (%)  ^2^Data do not sum to 1947 due to missing values  ^3^HIV- = HIV uninfected, HIV+ ART- = HIV infected not on ART, HIV+ART+ = HIV infected on ART, X^2^– test used to test relationships between categorical variables | | | | | |
